# The European multicenter trial on the safety and efficacy of guided oblique lumbar interbody fusion (GO-LIF)

**DOI:** 10.1186/1471-2474-11-199

**Published:** 2010-09-06

**Authors:** Christof Birkenmaier, Olaf Suess, Michael Pfeiffer, Ralf Burger, Kirsten Schmieder, Bernd Wegener

**Affiliations:** 1 Department of Orthopedic Surgery, University of Munich, Grosshadern Campus, Marchioninistr. 15, 81377 Munich, Germany; 2 Department of Neurosurgery, Charité Medical University Berlin, Campus Benjamin Franklin (CBF), Hindenburgdamm 30, 12200 Berlin, Germany; 3 Department of Orthopedic Surgery, Helios Rosmann Klinik Breisach, Zeppelinstrasse 37, Breisach 79206, Germany; 4 Department of Neurosurgery, Klinikum Nordstadt Haltenhoffstraße 41, 30167 Hannover, Germany; 5 Department of Neurosurgery, University Hospital Mannheim, Theodor-Kutzer-Ufer 1-3, Mannheim 68167, Germany

## Abstract

**Background:**

Because of the implant-related problems with pedicle screw-based spinal instrumentations, other types of fixation have been tried in spinal arthrodesis. One such technique is the direct trans-pedicular, trans-discal screw fixation, pioneered by Grob for spondylolisthesis. The newly developed GO-LIF procedure expands the scope of the Grob technique in several important ways and adds security by means of robotic-assisted navigation. This is the first clinical trial on the GO-LIF procedure and it will assess safety and efficacy.

**Methods/Design:**

Multicentric prospective study with n = 40 patients to undergo single level instrumented spinal arthrodesis of the lumbar or the lumbosacral spine, based on a diagnosis of: painful disc degeneration, painful erosive osteochondrosis, segmental instability, recurrent disc herniation, spinal canal stenosis or foraminal stenosis. The primary target criteria with regards to safety are: The number, severity and cause of intra- and perioperative complications. The number of significant penetrations of the cortical layer of the vertebral body by the implant as recognized on postoperative CT. The primary target parameters with regards to feasibility are: Performance of the procedure according to the preoperative plan. The planned follow-up is 12 months and the following scores will be evaluated as secondary target parameters with regards to clinical improvement: VAS back pain, VAS leg pain, Oswestry Disability Index, short form - 12 health questionnaire and the Swiss spinal stenosis questionnaire for patients with spinal claudication. The secondary parameters with regards to construct stability are visible fusion or lack thereof and signs of implant loosening, implant migration or pseudarthrosis on plain and functional radiographs.

**Discussion:**

This trial will for the first time assess the safety and efficacy of guided oblique lumbar interbody fusion. There is no control group, but the results, the outcome and the rate of any complications will be analyzed on the background of the literature on instrumented spinal fusion. Despite its limitations, we expect that this study will serve as the key step in deciding whether a direct comparative trial with another fusion technique is warranted.

**Trial Registration:**

Clinical Trials NCT00810433

## Background

For many degenerative, inflammatory and traumatic conditions of the spine as well as for spinal deformities, the definitive fusion of one or several spinal motion segments (spinal arthrodesis) remains the treatment of choice at this time. Fusion requires the preparation of a fusion mass between two vertebrae or their posterior elements and adequate stability for the period of time that is required to achieve solid bony bridging. While there are a number of different fusion techniques, the necessary stabilization is currently almost exclusively achieved by pedicle screw - rod - systems. This technique requires 2 pedicle screws to be placed into each vertebra, which for a single-level fusion translates to 4 screws and 2 interconnecting rods. Since for every individual screw placement there is an inherent risk of implant malpositioning and nerve injury, this particular risk is encountered 4 times in a typical single-level pedicle screw construct for spinal fusion. The typical implant-related complication rates for lumbar or lumbosacral spinal fusion surgery are reported as high as 19 percent [1,2]. On the background of such complication rates and also on the basis of biomechanical considerations [3], alternative trans-pedicular trans-discal fixation techniques have been tried. Such trajectories had already been used in combination with pedicle screw and/or transvertebral cage constructs for high dysplastic spondylolistheses [4-10]. Grob and recently Zagra (using the Grob technique) were the first to use 2 trans-pedicular, trans-discal screws alone to stabilize a lumbar or lumbosacral motion segment for posterolateral fusion [11,12]. While in theory this technique reduces the risk of screw misplacement and hence of nerve injury by 50 percent per fused motion segment, they still experienced some implant-related complications requiring revision surgery. These implant misplacements were judged to be due to the difficulty of drilling the anatomically demanding screw trajectories under fluoroscopic control alone. The "Guided Oblique Lumbar Interbody Fusion" (GO-LIF) procedure overcomes these problems by means of robotic-assisted computer navigation, whose accuracy has been established [13-15]. It also expands on the original Grob procedure in 3 important ways: First, it makes minimally invasive, percutaneous screw placement possible. Second, it allows for the combination with intervertebral cage fusion techniques. And third, it doesn't require the presence of spondylolisthesis. These 3 factors greatly expand the range of possible indications. However, with less than 20 cases having been performed worldwide, it cannot yet be known whether GO-LIF might in the future represent a valid alternative to pedicle screw-based stabilization techniques. This clinical trial was designed to examine the safety and the efficacy of the GO-LIF procedure.

## Methods/Design

This is a multicentric cases series that will be compared to literature control. The study design has been reviewed and approved by the ethics committee of the Ludwig-Maximilian-University of Munich, Germany. A participant insurance policy has been provided and the trial has been registered at http://www.clinicaltrials.gov[16]. The main author is responsible for the study design, for monitoring the data collection and the anonymization as well as for the data analysis. Independent experts in spinal surgery with no association to the GO-LIF procedure or the study have committed to evaluate and judge adverse events, should any occur. These experts would then report to the ethics committee as well as to the study leaders. There are 10 participating centers in Germany, Switzerland and Italy, where a total of 40 patients are to be recruited within 6 months. We plan to present descriptive statistics and to discuss the findings on the background of the published literature.

Included will be men and women between 18 and 80 years of age, capable of giving informed consent and with a clear clinical indication for monosegmental lumbar or lumbosacral fusion (with or without decompression) based on a diagnosis of at least one item in the following list:

• painful disc degeneration (black disc)

• painful erosive osteochondrosis

• segmental instability

• recurrent disc herniation

• spinal canal stenosis

• foraminal stenosis

Exclusion Criteria are:

1) Lumbar hyperlordosis >70° between the end plate of the lumbar vertebral body 1 and the end plate of the sacral vertebral body 1 (because of the risk of injury to the facet joint below the instrumented level).

2) Deformities of the vertebral bodies envisioned for instrumentation (or the sacrum).

3) Spondylolisthesis >grade 2 (Meyerding).

4) Scoliosis and other deformities in the coronal plane (not asymmetric disc space collapse).

5) Fractures of the vertebrae envisioned for instrumentation.

6) Osteoporosis or osteopenia (known diagnosis or as assessed by DXA or qCT).

7) Therapy with systemic corticosteroids or immunosuppressants.

8) Metabolic bone diseases, such as osteomalacia or Paget's disease.

9) Post inflammatory instability of the vertebral spine.

10) Status post radiation therapy of the relevant spinal region.

11) Current Coumadin (or Warfarin) or Heparin therapy for more than 6 months at the time of operation.

12) Malignant diseases with or without bone metastases.

13) Immunologic-inflammatory diseases (e.g. rheumatoid arthritis).

14) Diabetes mellitus.

15) ongoing infectious conditions.

16) body mass index (BMI) >30.

### Operative Technique

**Figure 1:**
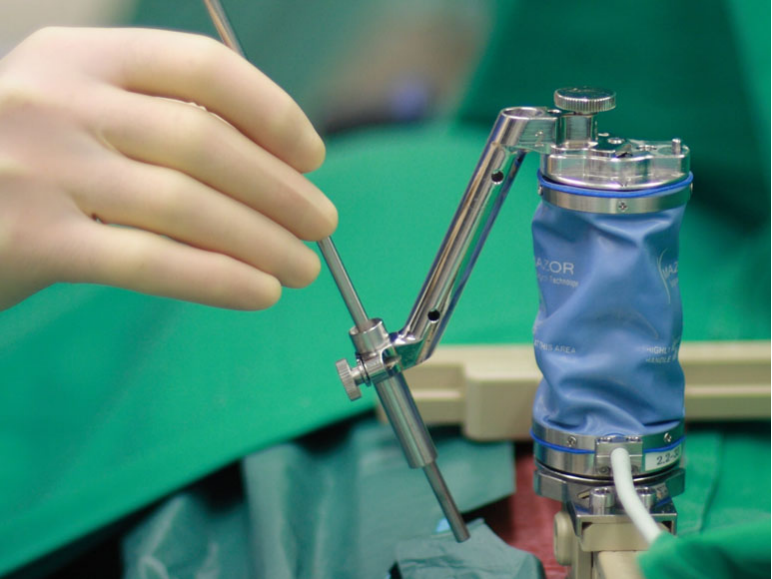
**The SpineAssist miniature robotic device is mounted on a radiolucent frame, which is attached to the patient**. One of the modular arms (arm #2 in this case) is attached to the miniature robotic device and carries the drill sleeve at its end. A Steinmann pin is manually passed through the drill sleeve for demonstration purposes.

**Figure 2:**
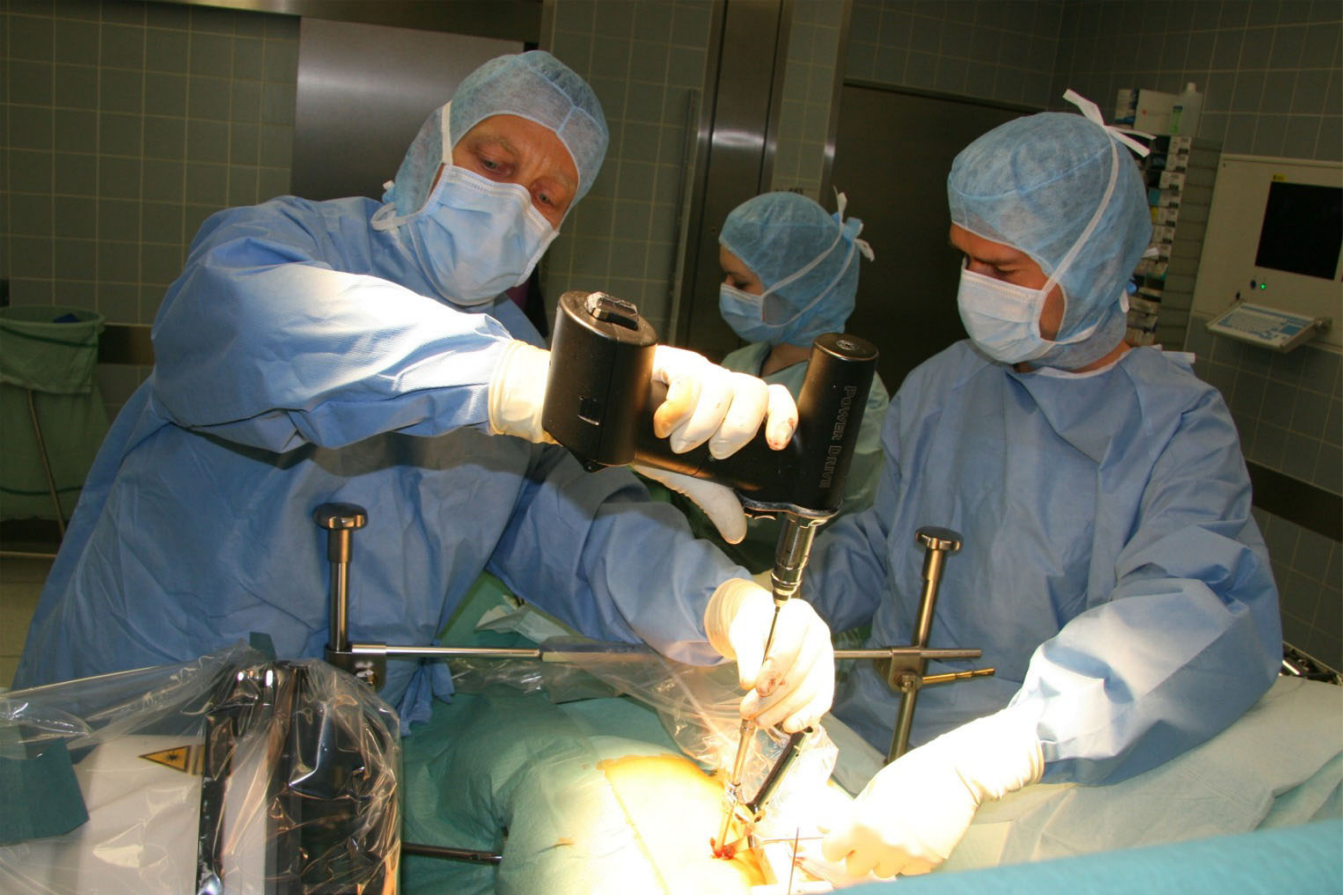
**Shows the percutaneous drilling of a GO-LIF screw trajectory, using the drill sleeve**. Lateral fluoroscopy is used to control for depth.

**Figure 3:**
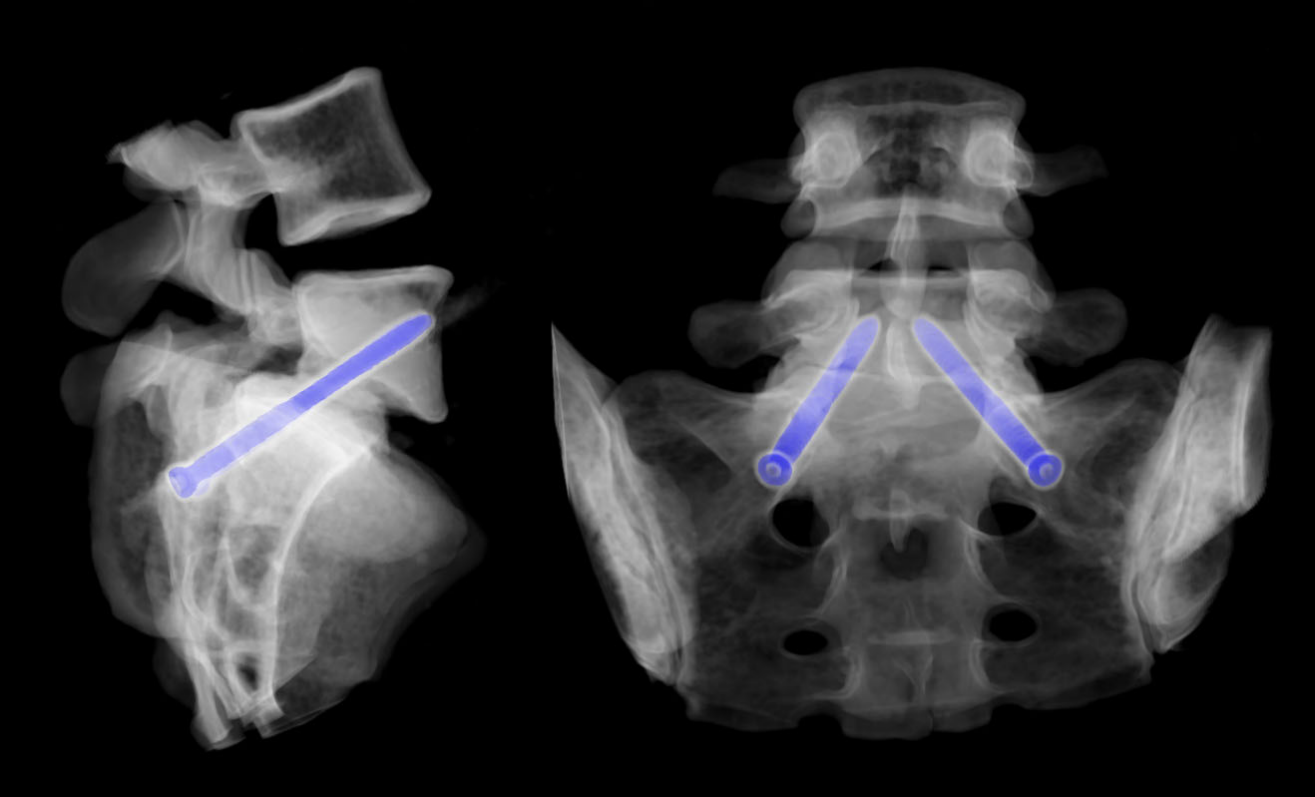
**3-D-reconstruction of a percutaneous in-situ fixation of a grade 2 isthmic spondylolisthesis at L5/S1 using the GO-LIF technique**.

The preoperative CT scan will be used for planning and navigation. In surgery, the SpineAssist miniature robotic device will be attached to the operating table and to the patient by means of a special bed frame and a Kirschner wire, which is anchored in a spinous process superior to the segment to be operated on (figure 1). After referencing and matching of the operative situation to the preoperative plan by means of 2 fluoroscopy images, the SpineAssist workstation will direct the miniature robotic device to the correct position. The miniature robotic device will then be fitted with one of three available arms (as prompted by the workstation), which carries a drill sleeve at its end. A support arm that is fixed to the bed mount will give additional stability so that the implant trajectory will be drilled in exactly the way it was planned (figure 2). If decompression is required, it will be performed according to microsurgical standard. In cases with a completely collapsed disc space, no interbody device is required and only bone graft may be used to fill the residual disc space if there is any. In every other case, an interbody fusion will be performed according to the surgical standards of a PLIF or TLIF procedure. Finally, the GO-LIF screw implants are placed and the procedure is ended (example shown in figure 3). While the example shown is a GO-LIF fixation at the L5/S1 level, GO-LIF can be performed at all lumbar levels. The GO-LIF fixation is not suited for spondylolisthesis reduction, which means that it can only be applied in situations of a spondylolisthesis where an in-situ fixation and fusion is planned.

The imaging modalities to be used in this study are plain radiographs and computed tomography (CT). The pre-operative CT, which is required to define the surgical anatomy, will be saved in such a way that it can also be used for the preoperative planning and the intraoperative navigation. A routine postoperative CT of the instrumented vertebrae will be acquired to evaluate implant positioning and to exclude intraspinal bleeding. This scan will also be used to compare the planned position of the GO-LIF implants to their real position and hence serve as a parameter for the safety of the procedure. Any implant deviations from the planned position will be measured and categorized in 1 millimeters increments. This data will be presented and discussed in full. Implants penetrating the pedicular or the vertebral cortex by more than 4 millimeters will be categorized as malpositioned according to the method by Gertzbein and Robbins [17]. Such implants will be considered unsafe in the sense of the target criterion. The plain radiographs will be used to assess construct stability, implant position, implant migration or loosening, formation of a sold fusion mass or indication of pseudarthrosis.

The following clinical data/scores will be evaluated:

• Current pre-operative analgesic treatment

• Visual Analog Scale for back pain (VAS back)

• Visual Analog Scale for leg pain (VAS leg)

• Oswestry Disability Index (ODI)

• Short Form - 36 health questionnaire (SF-36)

• Swiss Spinal Stenosis Questionnaire (SSS)

Data will be collected at the following measurement points:

1) Immediately preoperatively.

2) Intra- or perioperatively.

3) 6 weeks postoperatively.

4) 3 months postoperatively.

5) 6 months postoperatively.

6) 12 months postoperatively.

7) Thereafter, annually if after the completion of the proposed study, an extension of the follow-up period is applied for and approved.

The primary target parameters (to be assessed within the immediate perioperative phase) with regards to safety are:

• Number, severity, and cause of intra- and perioperative complications, in particular injury or irritation of nerve roots.

• Number of significant (>4 mm) penetrations of the cortical layer of the vertebral body or the pedicle by the implant, as recognized on a postoperative CT.

The primary target parameter with regards to feasibility (to be assessed within the immediate perioperative phase) is:

• Feasibility is defined as performing the intervention according to the preoperative plan. The number of interventions that could not be performed according to plan as well as the cause thereof serve as feasibility indicators.

The secondary target parameter with regards to construct stability (to be assessed on all follow-up visits until 12 months) is:

• In order to assess the stability of the GO-LIF fixation, the routinely performed functional radiographs after fixation operations will be evaluated.

The secondary target criteria with regards to clinical improvement (to be assessed on all follow-up visits until 12 months) are:

• VAS for back and leg pain, ODI and SF-36 health questionnaire and the SSS questionnaire.

We performed a power analysis in order to assess the required sample size to show safety with a power (1-β) of 0.8 and an α of 0.05. As basis for our calculation, we used the rates (5%-trimmed mean and standard deviation) of correctly placed pedicle screw implants in the best meta analysis available for such a purpose [18]. With an effect size (d) of 0.4478528 and an actual power (1-β) of 0.808629, the total sample size calculated as n = 33. Leaving some room for additional power and/or patients that might have to be excluded as a result of protocol violations, we decided upon n = 40 as the number of patients to be recruited.

## Discussion

This is a clinical case series without a direct control and hence without randomization, which brings certain limitations to our study. Our choice of study design was influenced by several considerations. For one, since this is the "phase 1" clinical study on a new fixation technique and therefore safety and efficacy are the primary parameters to be studied, we are at this point not primarily interested in showing superiority or non-inferiority of the GO-LIF technique as compared to any other established fusion technique. It would have therefore not strengthened this first study to include another fusion technique as a comparison. This even more so, as there is no universally accepted fusion technique that everyone would agree on as the gold standard against which to compare a new technique. The power calculation for this study was rendered difficult by the following factors. On the background of the very inhomogeneous literature on spinal fusion surgery and since we expect none or few neurological complications with the use of navigation, this parameter would not have been suitable for use in our power analysis. In view of our primary target criteria, we therefore had to use a technical parameter, in this case, the accuracy of screw placement. With various classifications and with the inconstant reporting of complications in clinical trials, we resorted to the data collected and evaluated by Kosmopoulos et al. as the basis for our power calculation [18]. Even though the number of patients to be included resulted from a power analysis, it should be remembered that based on Hanley's "rule of three", even if no adverse events are to occur it cannot be concluded that such events will not happen in future patients undergoing the same procedure [19]. Depending on the outcome of this study, a prospective and randomized trial comparing GO-LIF to another fusion technique will be the next step.

## Abbreviations

CT: computed tomography; DXA: dual energy Xray absorptiometry; GO-LIF: guided oblique lumbar interbody fusion; PLIF: posterior lumbar interbody fusion; qCT: quantitative CT; TLIF: transforaminal lumbar interbody fusion.

## Competing interests

Mazor Surgical Technologies Ltd. provides implants and navigation single-use items for the study cases at no charge to the participating institutions. Mazor Surgical Technologies Ltd. also provides an insurance policy for study participants. When needed, Mazor Surgical Technologies Ltd. supports surgical cases with navigation technicians. Beyond that, Mazor Surgical Technologies Ltd. does not financially support the study or any of the participating surgeons/investigators. No non-monetary benefits are provided. No participation fees of any sort are being paid to the included patients. The authors declare that they have no competing interests. Specifically, none of the authors has received beyond € 2000 for consulting or other scientific services from Mazor Surgical Technologies Ltd. from 2005 through 2010. None of the authors currently holds any shares/stock options in Mazor Surgical Technologies Ltd.

## Authors' contributions

CB is responsible for the study concept and design, the interpretation of the data and the manuscript draft. OS participates in the acquisition of data and in the critical review of the manuscript. MP is involved in study concept and design, he participates in the acquisition of data and in the critical review of the manuscript. RB is involved in the acquisition of data and in the critical review of the manuscript. KS participated in designing the study. BW is involved in concept and design, data analysis and the critical review of the manuscript.

All authors read and approved the final manuscript.

## Pre-publication history

The pre-publication history for this paper can be accessed here:

http://www.biomedcentral.com/1471-2474/11/199/prepub
